# Analysis of the diversity and tissue specificity of sucrose synthase genes in the long read transcriptome of sugarcane

**DOI:** 10.1186/s12870-019-1733-y

**Published:** 2019-04-25

**Authors:** Prathima P. Thirugnanasambandam, Patrick J. Mason, Nam V. Hoang, Agnelo Furtado, Frederik C. Botha, Robert J. Henry

**Affiliations:** 1 Centre for Plant Sciences, Queensland Alliance for Agriculture and Food Innovation, The University of Queensland, 306 Carmody Road, St. Lucia, Queensland 4072 Australia; 2 Crop Improvement Division, ICAR-Sugarcane Breeding Institute, Coimbatore, India; 3 Department of Plant Biotechnology, College of Agriculture and Forestry, Hue University, Hue, Vietnam; 4 Sugar Research Australia, Indooroopilly, Queensland 4068 Australia

**Keywords:** Sucrose synthase gene family, SuSy isoforms, Transcription, Sugarcane, Expression profiling, Sugarcane genes, Transcript diversity, Root tissue

## Abstract

**Background:**

Sugarcane accumulates very high levels of sucrose in the culm. Elucidation of the molecular mechanisms that allows such high sucrose synthesis and accumulation (up to 650 mM) is made difficult by the complexity of the highly polyploid genome. Here we report the use of RNA Seq data to characterize the sucrose synthase (SuSy) genes expressed in the transcriptome of the mature sugarcane plant.

**Results:**

Four SuSy gene families were identified in the sugarcane Iso-Seq long read transcriptome (SUGIT) through gene annotation of transcripts that mapped to reference SuSy genes from sorghum and maize. In total, 38, 19, 14, and 2 transcripts were identified for the four corresponding SuSy genes 1, 2, 4 and 7, respectively. Comparative studies using available SuSy genes from sorghum (1, 2, 4, 6, 7) and maize (1–7) revealed that the sugarcane SuSy genes were interrupted by multiple introns and that they share a highly conserved gene structure. Spatial expression of the four SuSy genes in sugarcane genotypes and in the progenitor species, *Saccharum spontaneum* and *Saccharum officinarum*, was studied in the leaf and root tissues and also in three regions of the culm tissue; top, middle and bottom internodes. Expression profiles indicated that all SuSy transcripts were differentially expressed between the top and bottom tissues, with high expression in the top tissues, lower expression in the bottom and moderate expression in the middle, indicating a gradient of SuSy activity in the sugarcane culm. Further, the root tissue had similar expression levels to that of the top internodes while leaf tissues showed lower expression. In the progenitors, SuSy7 was found to be highly expressed in *S. officinarum* while the other three SuSy genes had moderate expression in both the progenitors.

**Conclusions:**

The high expression of the SuSy genes in sink tissues, the top internodes and the roots suggests functional roles in sucrose utilization to support growth. The SuSy7 gene has not been previously reported in sugarcane. As sugarcane is unique in storing such high amounts of sucrose, it is possible that there are more SuSy genes/isoforms with specific expression patterns to be discovered in this complex system.

**Electronic supplementary material:**

The online version of this article (10.1186/s12870-019-1733-y) contains supplementary material, which is available to authorized users.

## Background

Sucrose synthase (SuSy) is widely considered as a key enzyme involved in sucrose metabolism and is probably important in carbon partitioning towards polysaccharide synthesis, sucrose turnover and an adenylate-conserving pathway of respiration [1]. Cell wall formation is inhibited in SuSy mutants in maize [2, 3], antisense carrot plants [4] and cotton seeds [5]. However, the universal role of SuSy is still unclear as normal growth occurs in knock down mutants of Arabidopsis plants [6].

Several paralogous genes of SuSy have been identified and characterized in multiple plant genomes [7–12], while very limited information on SuSy genes is available for sugarcane [13–18]. SuSy catalyzes the reversible conversion of sucrose into fructose and UDP-glucose, which are the primary substrates for respiration, starch and cell wall constituents [1, 19] and fibre development [20]. SuSy is reported to be active in young internodes of sugarcane stems [16, 21]. It is also found to be more highly expressed in high sugar genotypes than in low sugar genotypes [22] wherein high levels of SuSy activity was correlated with an increase in sucrose accumulation rate and ripening. However, there are contradictory observations made in other studies (i.e. in [13, 23]) indicating no correlations between SuSy and sucrose accumulation. The reaction catalyzed by SuSy is readily reversible, and the enzyme is said to be functioning primarily in the direction of sucrose degradation (sucrose cleavage into glucose and fructose subunits) to provide sugar nucleotides for glycosylation of varied molecules. SuSy is highly correlated with sink strength in various crops like potato [24], carrot [25, 26], maize [8] and pea embryos [27]. In addition, SuSy activity is found to be associated with sugar import [28], organellar function [29] stomatal function [30], plant’s response to environmental stresses and nitrogen fixation [31]. There are studies on the association of SuSy with cellulose synthases forming complexes to channel UDP-glucose towards cellulose biosynthesis [19].

The identification and subsequent characterization of different SuSy genes forms the basis for understanding their roles in the physiological, metabolic and molecular mechanisms of different growth processes in plants. At least three SuSy genes are thought to be found in most plant species encoded by a small multigene family. There are six distinct active SuSy genes in Arabidopsis [10, 32] and model legume *Lotus japonicus* [33]. Rice is reported to have seven SuSy genes [34] and was previously thought to have six genes [10]. Similarly, diploid cotton was reported to have seven SuSy genes [7], which was later updated to eight [20, 35]. The pea has three SuSy genes [27]. A recent genome wide study in grapes reported five SuSy genes [36]. Maize has three distinct genes, *Sh*1*, Sus*1 and *Sus*3 [9, 37]. However, the NCBI-GenBank has seven SuSy genes (1–7) sequences for *Zea mays* (*Zea mays* annotation version 101 v 7.3, released March 2017). Similarly, the *Sorghum bicolor* annotation release 101v 7.4 (released June 2017) has SuSy genes 1, 2, 4, 6, and 7. *Nicotiana tabacum* is reported to have 14 SuSy genes [38]. The tetraploid cotton, *G. hirsutum* has the largest SuSy family to date, containing 15 SuSy genes [35]. SuSy genes have high structural similarity, functional diversity and are reported to have tissue specific expression during various stages of plant development. Tissue- and development stage- specific expression of SuSy is also reported in rice [10], carrot [39], poplar [40], cotton [7], maize [9, 41], sugarbeet [42] and citrus [43] implying that each SuSy gene may have a distinct role at a specific developmental stage in a specific tissue.

The sugarcane culm and roots are the major sinks in sugarcane [44]. There are two clear phases of internode growth in the culm. Using a base temperature of 18 °C internode elongation stops after 150°Cd (heat units) while dry matter accumulation continues for up to 800°Cd [45, 46]. By the time internode length stopped increasing, the dry weight of the internodes was still less than half its final value [45, 46]. The pattern of carbon partitioning in the culm changes significantly during the elongation and biomass accumulation phases of the internode [47, 48]. The water-insoluble component (primarily cell wall), and non-sucrose-water-soluble fraction (reducing sugars, amino acids and organic acids), represents approximately 90% of the total dry matter during the internode elongation phase (up to internode 5). Sucrose accumulation starts in the young internodes but accelerates sharply when internode elongation stops. In internode 10, 50% of the dry mass is sucrose and this elevated sucrose comes at the expense of the other water solubles and fibre.

Sucrose synthesis in sugarcane is a continuous process and sucrose accumulation in the culm is initiated after internode elongation has stopped [14], takes place after six months of crop growth from the time of planting, coinciding with the development of stem and its elongating internodes [49] . The parenchymatous cells initially store sucrose in the vacuole which is a reversible process depending on the growth and developmental conditions [50], but in the mature culm up to 30% of the sucrose can be in the apoplast [51]. Many genes, including the SuSy genes, have been proposed to be involved in controlling sucrose synthesis and accumulation. In a few plant species such as *Arabidopsis*, SuSy genes have been well studied, however, in sugarcane, our understanding of SuSy genes still limited. Due to the importance of sucrose in sugarcane, the SuSy genes, especially their identity, gene structure, evolutionary mechanisms and potential functions in sugar and fibre synthesis and accumulation, needs to be well explored. Previous studies suggested the presence of a small gene family encoding different SuSy isoforms within the polyploid sugarcane, but to date characterization of the SuSy genes have not been reported, except for SuSy1 and 2 [15] and SuSy4 [52]. Recently a haplotype identification study of SuSy genes has reported five genes (1–5) in sugarcane [18]. With advances in sequencing technology, RNA sequencing (RNA-Seq) has become an effective and powerful tool for transcriptome analysis, that includes quantifying gene expression/allele-specific expression, discovery of novel transcripts and alternatively spliced genes [53].

In the current study, we report the identification and characterization of four SuSy genes (1, 2, 4 and 7) expressed in the sugarcane transcriptome derived from various tissues of a mature crop (at 10 months after planting) and their expression patterns at the transcriptome level. In addition, a set of transcriptomes (RNA-Seq read data) from three different regions of the culm (mainly top, middle and bottom internodes), leaves and root tissues from another independent experiment (Mason et al., unpublished) was utilized for checking the tissue specific expression of the four SuSy genes identified from the sugarcane transcriptome. The results presented in this work provide new insights into the functional diversity of the sugarcane SuSy gene family in response to growth and development and most importantly, sucrose synthesis and accumulation as the crop matures and stores maximum levels of sugar at this growth stage. The analyses in this study mainly focused on the gene identification, exon/intron organization, evolutionary relationship, and tissue-specific expression patterns of the sugarcane SuSy genes identified. Sugarcane is an autopolyploid with each locus having multiple haplotypes from 8 to 14 which is an indication of the level of heterozygosity that is likely to have contributed to the high biomass yield of sugarcane [18, 54]. As sugarcane is unique in storing such high amounts of sucrose, it is possible that there are more SuSy genes/isoforms with specific expression patterns suggesting the need for further studies. A new isoform SuSy7 is reported for the first time in sugarcane bringing the number of SuSy members from 5 [55] to 7, although SuSy 3, 5, 6 were not found in our study.

## Materials and methods

### Database search and bioinformatics analysis

A search for SuSy genes in the sugarcane Iso-Seq transcriptome database SUGIT, previously reported in [56], was performed in order to identify all members of the SuSy gene families. The strategy used to obtain the available members of SuSy gene family in the transcriptome was as follows. Using CLC Genomics Workbench version 10 (CLC-GWB, CLC Bio-Qiagen, Aarhus, Denmark), the SuSy gene sequences from *Sorghum bicolor* and *Zea mays* were used as query to search against the SUGIT database. Sequences of some of the SuSy genes from *Arabidopsis,* rice*,* wheat and bamboo were also used as queries in order to obtain a comprehensive list of putative SuSy genes from the transcriptome. All sequence data used in this study were collected using the keyword “sucrose synthase” in the NCBI-GenBank and UniProt databases. Initially, the sequences were mapped using the large gap mapping tool in CLC-GWB with length fraction (LF) (0.8) and similarity fraction (SF) of (0.5) in order to retrieve the sequences with less stringency followed by a LF of 1 and SF of 0.9 with the retrieved reads with their respective reference SuSy genes. Other bioinformatics analyses, such as amino acid composition and conserved domains of the SuSy genes were performed using the Expert Protein Analysis System (ExPASy) (http://www.expasy.org/tools/ protparam.html). Substitutions per synonymous site (Ks) and the non-synonymous divergence (Ka) values for each gene among the transcripts were calculated using the Nei-Gojobori method [57] implemented in MEGA v.7 [58].

### Gene structure and phylogenetic relationship analysis

The alignment of SuSy genes with references were performed using Geneious v.9 (https://www.geneious.com/). The schematic structures of SuSy genes, based on exon/intron data, were produced using Geneious v.11 with the reference gene sequences. The genomic location of each SuSy gene was determined using GBrowse and BLAST tools based on scaffold information for *Sorghum bicolor* available from Phytozome v12.1*.* Predicted conserved domains were screened within the deduced amino acid sequences of corresponding SuSy genes using the InterProScan web server (https://www.ebi.ac.uk/interpro/) and PROSITE (https://prosite.expasy.org/). The MEME suite was used for predicting motifs on the SuSy amino acid sequences (http://meme-suite.org/tools/meme). The parameters were set as follows: zero or one occurrence of a single motif per sequence; 30 as the maximum number of motifs to find. All other parameters were set at default. The open reading frame (ORF) lengths of the genes were analysed using the ORF Finder in NCBI. A phylogenetic tree was generated using the full-length protein sequences of the SuSy genes of sorghum, rice and maize along with the sugarcane SuSy genes 1, 2, 4 and 7. A total of 24 sequences were used for phylogeny the details of which are listed in the Table 1. Multiple alignment of the nucleotide and deduced amino acid sequences were performed using the programs ClustalW available in the MEGA 7.0 phylogeny program (http://www.megasoftware.net) with default parameters. The phylogenetic tree of deduced SuSy proteins was constructed by neighbour-joining algorithm with a bootstrap of 1000 replicates [59].

**Table 1:** Sequences used for phylogenetic analyses from sugarcane, sorghum, rice and maize

Gene	Sugarcane		Sorghum		Maize		Rice	
	A.A	Accession	A.A.	Accession	A.A.	Accession	A.A.	Accession
Susy1	816	–	816	XP021305168	816	NP001105411	816	AEX32874
Susy2	802	–	802	XP021312610	802	NP001105323	808	AAL31375
Susy3	–	–	–	–	809	AAM89473	816	ABL74561
SuSy4	809	–	809	XP002465303	809	XP008655408	809	AEX32877
SuSy5	837	AGT16515	–	–	852	A0A1D6GWZ8	855	AEX32878
SuSy6	–	–	863	XP021315397	849	XP008679107	857	XP015626470
Susy7	857	KF184934	855	XP021305179	857	XP008645119	855	AEX32880

A.A. – protein length in amino acids; Accession -NCBI-GenBank accession numbers

### Tissue specific expression of SuSy genes

The tissue-specific expression profiles of SuSy genes 1, 2, 4 and 7 were examined in sugarcane hybrid genotypes Q208^A^ and KQ228^A^. KQ228 is a commercial, high yielding, early to mid-maturing cane, developed by Sugar Research Australia (SRA); parentage Q135 x QN62–1232. Q208 is a commercial, high yielding cane, with moderate tolerance to herbicides, this cane was also developed by SRA: parentage QN80–3425 x CP74–2005. Expression profiles of the SuSy gene transcripts (1, 2, 4 and 7) were defined using a set of RNA-Seq data derived from leaves 1 and 5 (where leaf 1I is the first visible dewlap), top (3–5), middle (10–12) and bottom (lowest 4) internodes, and root tissue. For leaf samples, the midribs were removed and leaf lamina cut into segments. Internode samples from harvested culms were immediately cut into 0.5 cm-thick slices, followed by the removal of the rind and diagonal separation of the remaining pith into small 0.5 cm cubes, using a pair of secateurs. Internode samples were collected in internodal regions of 3 samples, which were bulked following extraction. The immature and mature root samples were collected from potted sugarcane plants. All the samples were snap-frozen in liquid nitrogen, and stored at -80 °C until RNA extraction. In total, 6 (root), 12 (leaf) and 54 (internode) samples were processed. After bulking there were a total of 36 RNA samples (18 internode, 12 leaf and 6 root). RNA extractions were conducted using the combined Trizol kit and RNeasy Plant minikit methods as described in [60] and checked for purity using an Agilent chip and Agilent Bioanalyser 2100 (Agilent Technologies, USA). Sufficient purity was obtained in all tissues for Illumina RNA-Seq application. RNA Seq was performed using an Illumina HiSeq 2000 at the Queensland Brain Institute, University of Queensland, Australia. Three replicated individual RNA-Seq reads were obtained for each tissue. Read adapters and quality trimming and all other downstream processes were performed in CLC-GWB with a quality score limit < 0.01 (Phred Q score ≥ 20), allowing a maximum of two ambiguous nucleotides, and removing reads below 75 bp. Paired end reads were counted as a single read in the RPKM algorithm. Details of RNA-Seq reads from various tissues are given in the Additional file 1: Table S1. For obtaining the expression values for individual SuSy genes in RPKM, RNA-Seq analyses were performed using the transcriptome reference database SUGIT with 0.8 and 0.8 as LF and SF settings respectively for each of the three replications for a sample. The experiments were carried out individually and the RPKM values for SuSy transcripts were obtained from each tissue and each replication, from the normalized reads of 40 million across the samples (as the lowest among the read counts, Additional file 1: Table S2) to avoid biases and make the expression values comparable across the tissues. In addition, the expression of the SuSy genes was checked in culm transcriptomes (top, middle and bottom pooled) from the progenitors *S. spontaneum* and *S. officinarum,* from another study [61] and in a set of high and low sugar genotypes (14 genotypes, differing in sugar and fibre contents) [62]. For visualizing of the expression pattern of SuSy genes, the average RPKM values of three replicates **(**Additional file 1: Table S2**)** were analysed in Microsoft Excel 2013. The heat maps of SuSy gene expression were generated by Pheatmap v1.0.8 R package [63, 64] using the log2-scaled (RPKM+ 1) values. The expression data is further validated using One way ANOVA and Tukey’s tests available from the SPS stats v.23 and heat maps were generated for the FDR corrected *p*-values.

### SuSy gene analysis using the draft reference genome from *Saccharum* hybrid cultivar SP80–3280

The draft reference genome from *Saccharum* hybrid cultivar SP80–3280 which is available from the NCBI database under the BioProject accession PRJNA272769 [65],was used as a reference genome for the SuSy transcript read mapping. Mapping settings were 0.5 to 0.8 for the similarity fraction and 0.8 for the length fraction. These settings were used to find the genomic sequences for other SuSy genes, present if any.

## Results

### Identification of SuSy genes in sugarcane transcriptome

**Fig. 1:**
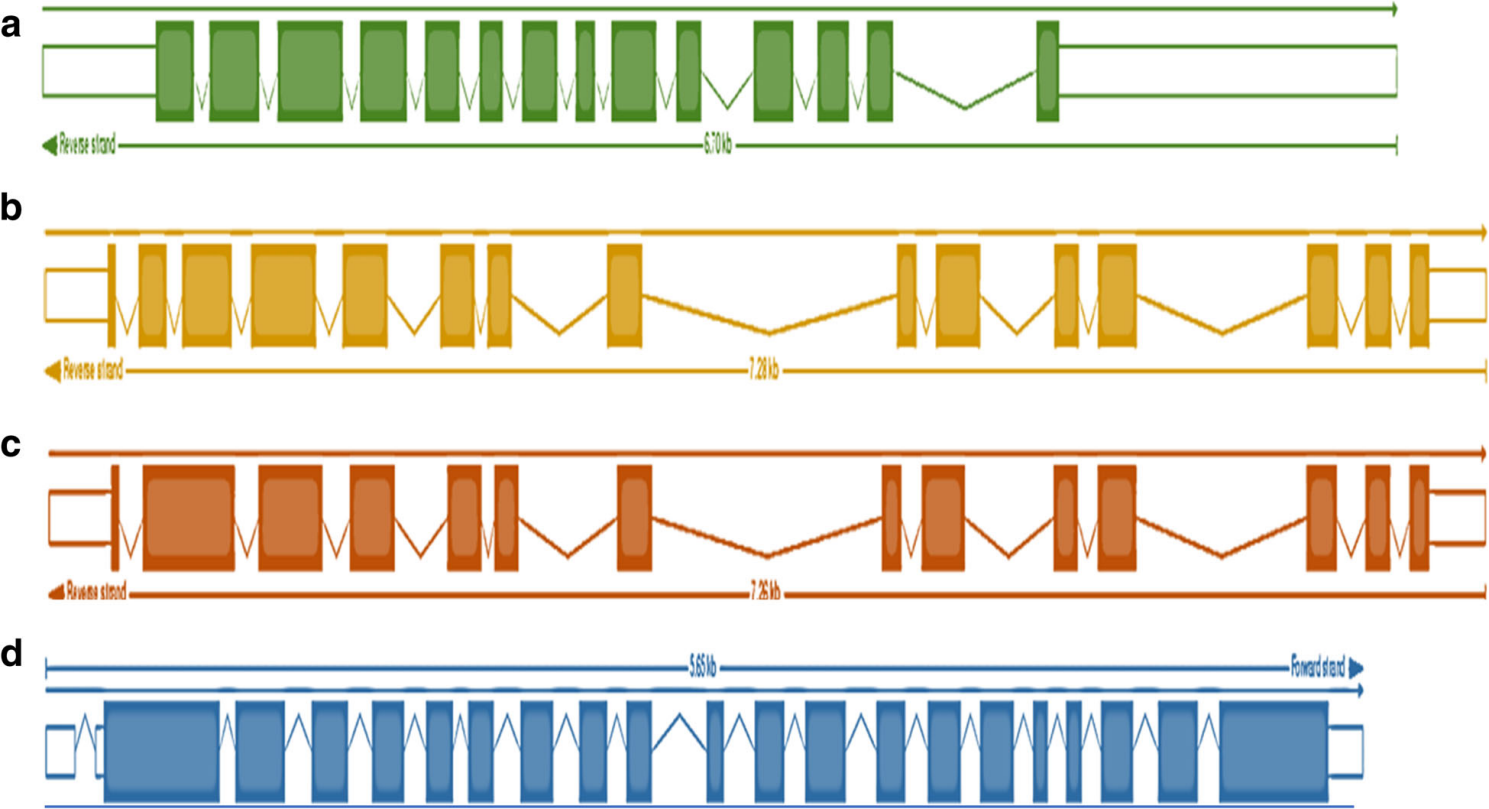
Schematic representation of exon-intron structure of the four SuSy genes identified from the sugarcane transcriptome SUGIT. Using Blast in the Ensembl plants database with *Sorghum bicolor* and *Zea mays* as reference genomes, the gene structures were predicted. The boxes and lines represent exons and introns respectively and the empty boxes represent UTR regions. **a** SuSy1, **b** SuSy2, **c** SuSy4 and **d** SuSy7

**Fig. 2:**
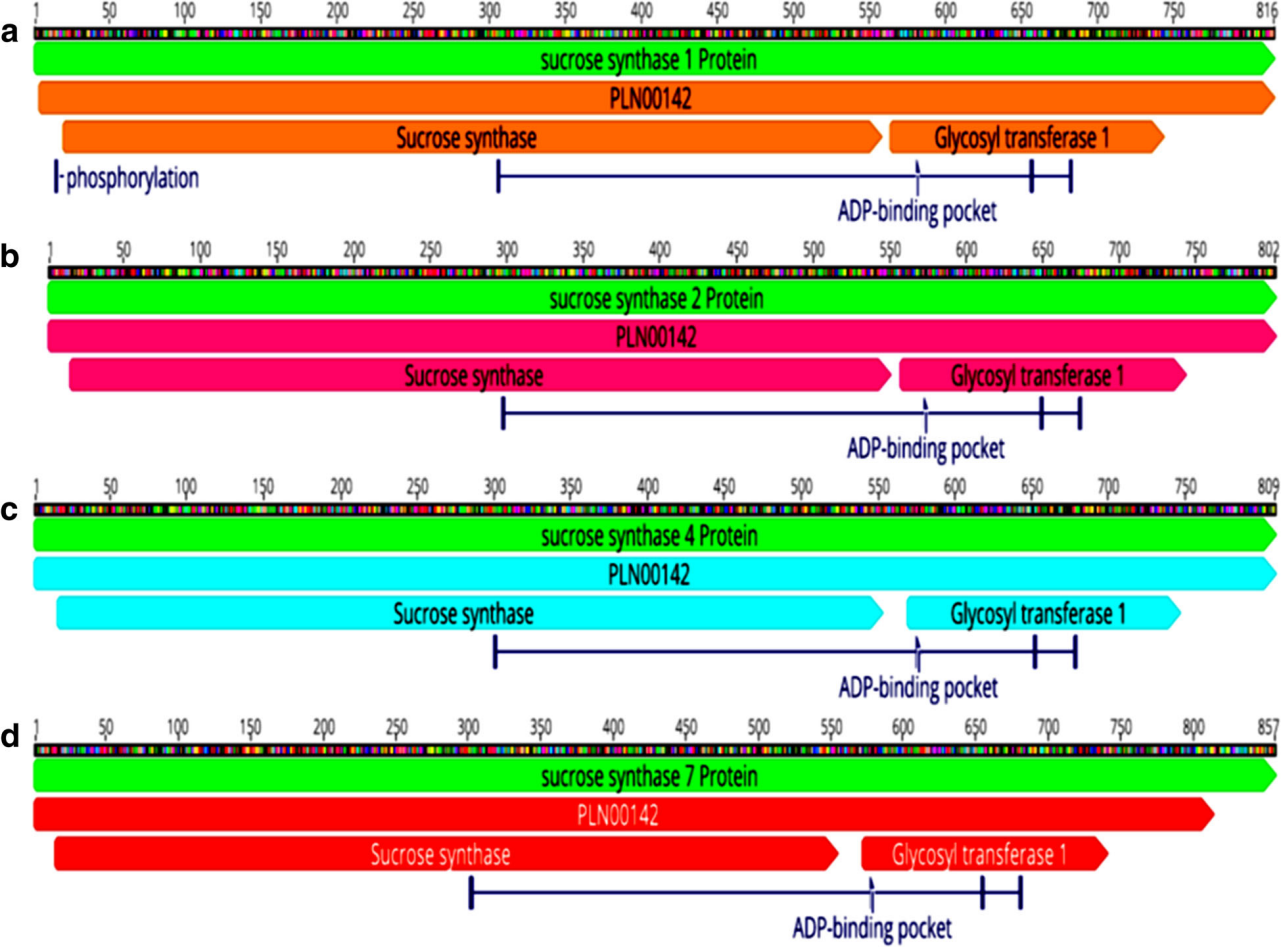
ORF and conserved domain prediction on SuSy transcripts using Geneious v11. **a**) SuSy1, (**b**) SuSy2, (**c**) SuSy4 and (**d**) SuSy7

**Fig. 3:**
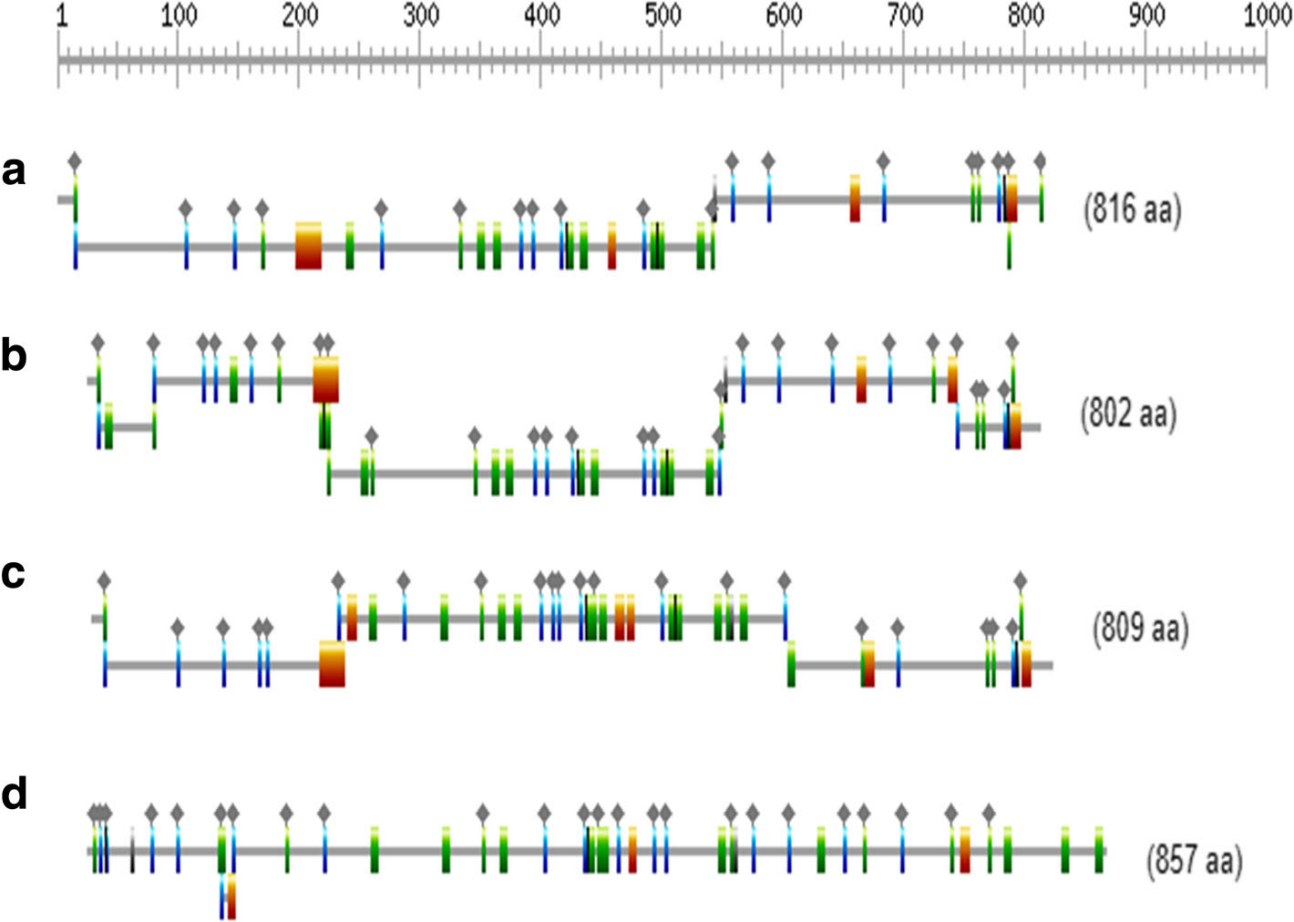
Graphical representations of domains displayed on the four different SuSy protein sequences using ScanProsite. The orange box indicates leucine zipper (PS0009) (in **a**-**c**), while green bars indicate Myristyl (myristolylation sites) (PS00008), orange bars indicate Tyrosine kinase phosphorylation sites, and blue bars indicate Casein kinase II phosphorylation sites (PS00006). **a**) SuSy1, (**b**) SuSy2, (**c**) SuSy4 and (**d**) SuSy7

From the sugarcane transcriptome reference database SUGIT, 74 transcripts matching with the SuSy reference genes were retrieved **(**Additional file 1: Table S3; Additional file 2: Figure S1). They were classified into four groups, depending upon the sequence similarity. Four full-length genes of sugarcane SuSy genes SuSy1 (transcript contig c109934f1p24379), SuSy2 (transcript contig c99109f1p142868), SuSy4 (transcript contig c81016f2p32950) and SuSy7 (derived from transcript contigs c96232f1p0928 and c65347f1p02616) were identified for gene structure, comparative and phylogenetic studies. Using a BLAST search against the sorghum genome, it was found that SuSy1 and 4 are located on chromosomes 1, while SuSy2 and 7 are on chromosome 10 (Table 2). The individual SuSy gene transcripts (one full-length transcript for each gene is described here, although variations might be present within the transcripts for each gene. For example, the 19 transcripts for SuSy1 gene were 95.22–100% similar to each other. The molecular weights of the proteins encoded by SuSy genes 1, 2, 4 and 7 were 93, 91, 96 and 97 kDa, respectively. SuSy2 was the highly represented gene with 38 transcripts while there were only two transcripts of SuSy7. There were about 19 and 14 transcripts of SuSy1 and SuSy4 respectively. Only for SuSy2, reference genomic sequence from sugarcane was available to infer the exon -intron structure, while for others, reference sequences from *Sorghum* and *Zea mays* were utilized. Each gene had a distinct structure with regard to intron and exon placement (Fig. 1a-d). The results obtained from NCBI-CDD search and InterProScan tools indicated two domains; the glycosyl transferase (GT1) and sucrose synthase domain (SS) characteristic of SuSy proteins. However, these vary in position among different SuSy proteins (Fig. 2a-d**)** and PROSITE domains distribution throughout the full-length protein sequences (Fig. 3a-d). The alignment of all the four SuSy gene transcripts with reference genes are shown in the Additional file 2: Figure S2, S3, S4 and S5.

**Table 2:** Characterization of the four SuSy genes identified in the SUGIT transcriptome database

Gene	Chromosome (with respect to Sorghum)	Molecular weight (Da)	pI	Protein length (aa)	Transcript length (bp)
Susy 1	1	93,767.18	6.13	816	2409
Susy2	10	91,766.90	5.82	802	4652
Susy4	1	96,244.03	6.88	809	2950
Susy7	10	97,791.07	7.80	857	2632

### SuSy1

There were 19 transcripts matching to SuSy1 reference genes. These transcripts ranged from 909 to 4438 bp in size. The deduced protein was 816 amino acids in size corresponding to the coding sequence of 2448 bp. There were two domains, SS domain spanning from 20^th^ amino acid isoleucine to the 558^th^ amino acid tyrosine. The GT1 spanned from 283^rd^ asparagine till 766^th^ methionine. The ATP binding domain was present between 306^th^ glycine till 683^rd^ threonine. SuSy1 showed 99% similarity with reference genes from sugarcane (AGI56230.1), sorghum (XP_002465161.1) and maize (XP_008659017.1).

### SuSy2

There were 38 transcripts matching to SuSy2 reference genes. These transcripts ranged from 409 to 4652 bp in size. The deduced protein was 802 amino acids in size corresponding to the coding sequence of 2406 bp. The SS domain began from 15^th^ amino acid leucine to the 550^th^ amino acid tyrosine. The GT1 domain spanned from 275^th^ asparagine till 758^th^ methionine. The ATP binding domain was present between 297^th^ glycine till 675^th^ threonine. The full-length reference gene for *Saccharum* SuSy2 in NCBI is 7771 bp longer with 1–16 exons (accession number AY118266). About 20 SuSy2 transcripts were found to have 16 exons, and few of them were found to retain introns in certain cases, when aligned with the full-length *Saccharum* SuSy2 reference gene.

### SuSy4

There were 14 transcripts matching to SuSy4 reference genes. These transcripts ranged from 501 to 3255 bp in size. The deduced protein size was 809 amino acids corresponding to the coding sequence of 2428 bp. The SS domain was found to be located from 16^th^ amino acid valine to the 553^rd^ amino acid tyrosine. The GT1 spanned from 277^th^ asparagine till 763^rd^ methionine. The ATP binding domain was present between 301^th^ glycine till 679^th^ threonine.

### SuSy7

There were 2 transcripts matching to SuSy7 reference genes. One of the transcripts was 928 bp while the other was 2632 bp in size. The protein size was 857 amino acids corresponding to the coding sequence of 2568 bp. The SS domain spanned from 17^th^ amino acid methionine to the 558th amino acid tyrosine. The GT1 domain spanned from 283^rd^ asparagine till 767^th^ valine. The ATP binding domain was present between 306^th^ glycine till 685^th^ threonine. The full-length sequence information for the SuSy7 protein was derived from two transcripts (the longer transcript was short of the initial 53 amino acids and the other transcript was incomplete in the 3′ end).

### Comparative studies of SuSy genes in sugarcane, sorghum and related genera

**Fig. 4:**
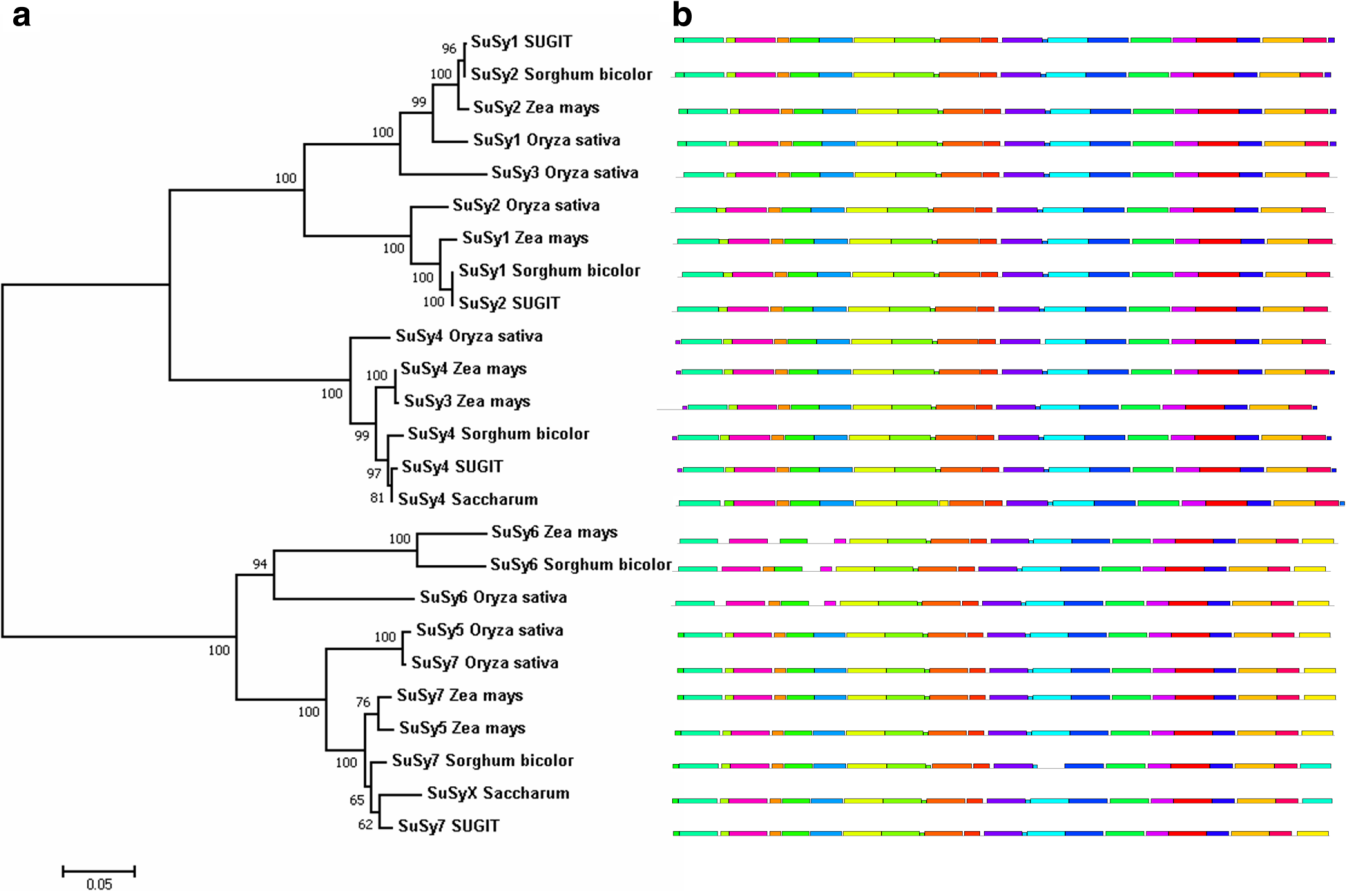
Phylogenetic tree and distribution of conserved motifs of SuSy proteins. **a** Phylogenetic tree of SuSy proteins from *Saccharum* hybrid *O. sativa*, *S. bicolor*, and *Z. mays*. This tree was constructed using MEGA 7.0 program by the N-J method with 1000 bootstrap replicates based on amino acid sequence. The tree is divided into two clades (clades I, II). The clade I is further divided into two sub-clades suggesting the three classes of SuSy genes reported in angiosperms. **b** Distribution of conserved motifs predicted using MEME program with a motif limit of 30 for distinguishing the SuSy genes

The Ka/Ks ratios for the transcripts of SuSy genes were < 1 revealing that these genes evolved under negative or purifying selection (Additional file 1: Tables S4, S5 and S6). To study the sequence similarity and evolutionary relationship among the SuSy gene family members in sugarcane, a phylogenetic tree was generated using the full-length protein sequences of the SuSy genes from sorghum, rice and maize along with the sugarcane SuSy genes 1, 2, 4 and 7 (Fig. 4a). The MEME suite identified motif positions for each member that were highly conserved without any insertions or deletions **(**Fig. 4b**)** when the motif to be predicted was set below 25. However, a motif prediction for 30 resulted in minor variations that helped differentiate among the gene families. As expected **t**he phylogenetic tree revealed a close relationship among the orthologous SuSy gene families. There were two distinct clades with SuSy1, 2, 3, 4 forming one clade, and SuSy5, 6 and 7 forming another. In addition, the conserved exon-intron structures of the SuSy genes across the monocots species available in Phytozome using GBrowse-BLAST tool with sorghum genome as the reference were shown in Additional file 2: Figure S6.

### Expression profiling of SuSy genes in various tissue transcriptomes

**Fig. 5:**
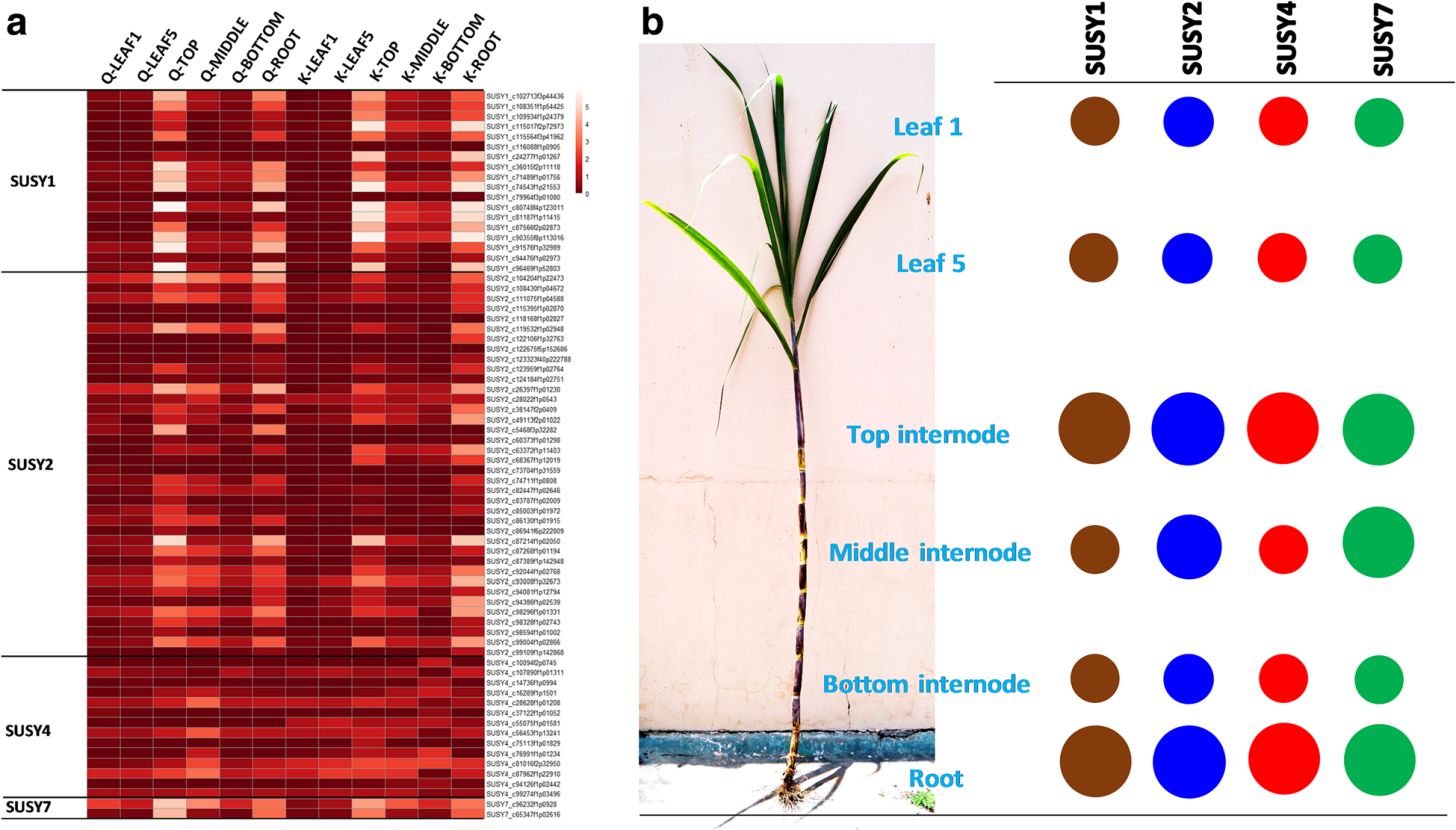
Tissue specific expression profiles of SuSy genes. **a** Heat maps showing log2-scaled RPKM values for the tissue specific expression profiles of SuSy genes 1, 2, 4 and 7 in the sugarcane hybrid genotypes Q208^A^ and KQ228^A^ denoted by Q and K, respectively for SuSy1, SuSy2, SuSy4, and SuSy7. RNA-Seq reads from leaf 1, leaf 5, (1st visible dewlap leaf and 5th leaf), top, middle and bottom (denoting the three different regions of internode/culm), and root tissues were used for obtaining the expression values of the four SuSy genes in RPKM. **b** A schematic representation of the over-all pattern of SuSy gene expression in different tissues

The expression values (log2- RPKM values) were obtained for the four genes and heat maps were generated to visualize the expression profiles (Fig. 5a and b). All four isoforms were highly differentially expressed between the top and bottom internodes with the top internode having very high expression of SuSy genes, while the bottom showed very low levels of expression. The middle internode had moderate expression, thus indicating a gradient of SuSy expression with high, moderate and low expression in top, middle and bottom internodes respectively. However, the expression of SuSy4 in the middle internode was found to be higher than the top and bottom internode (genotype Q208^A)^ or equal to the top and bottom internodes (genotype KQ228^A^**)**. The leaves showed very low expression levels for all the four SuSy genes while the roots displayed very high levels similar to that of the top internode. The top internode had the highest expression levels of SuSy genes 1, 2 and 7, followed by the root tissues, while the middle internode showed high expression levels for SuSy4 only in genotype Q208. In KQ228 genotype there was also significantly higher expression of SuSy7. The expression profiles were mostly consistent in both the genotypes Q208^A^ and KQ228^A^, across all the tissues used for the analyses and were statistically validated (Additional file 1: Tables S7, S8; Additional file 2: Figure S7, S8).

**Fig. 6:**
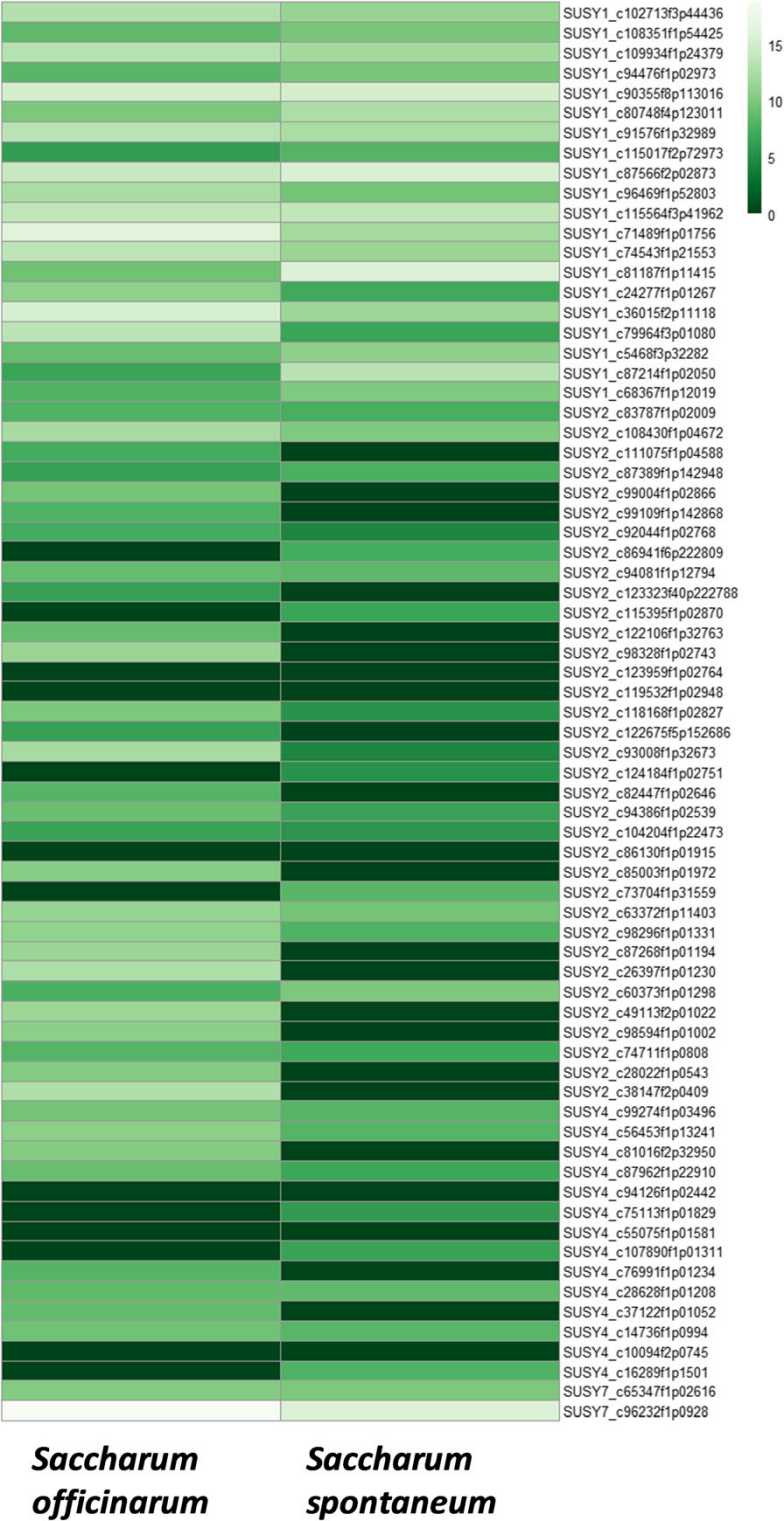
SuSy genes expression in the progenitor transcriptomes, *S. officinarum* and *S. spontaneum* visualized using log2-scaled RPKM values in heat maps. The transcript (c96232f1p0928) showing highest expression is annotated as SuSy7 which has a probable sub-genomic specific expression from *S. officinarum*. There were few *S. spontaneum* specific SuSy1 transcripts (c81187f1p11415, c87566f2p02873, c90355f8p113016) and *S. officinarum* specific SuSy1 transcripts (c36015f2p11118, c71489f1p01756)

In case of the progenitor species *S. officinarum* and *S. spontaneum*, the expression of SuSy transcripts is shown in the Fig. 6. SuSy7 (c96232f1p0928) transcript showed highest expression in *S. officinarum*. The other SuSy7 transcript (c65347f1p02616) also showed high expression in *S. officinarum*. There were few *S. spontaneum* specific SuSy1 transcripts (c81187f1p11415, c87566f2p02873, c90355f8p113016) and *S. officinarum* specific SuSy1 transcripts (c36015f2p11118 and c71489f1p01756). In general, SuSy2 transcripts were found to be highly expressed in *S. officinarum* than *S. spontaneum*.

### **SuSy gene analysis using the sugarcane** SP80–3280 **reference genome**

Using reference genes from sorghum and maize, the genome sequences of the sugarcane cultivar SP80–3280 was checked for the presence of the full-length sequences for SuSy genes 3, 5 or 6. When the SuSy5 sequence from *Zea mays* was used for mapping, two genomic reads (JXQF01182951 and JXQ01195768) mapped with a consensus of 504 and 2104 respectively. This sequence was found to have 60% query coverage and 98% similarity with SuSy7 and 51% query coverage and 100% similarity with SuSy5.

## Discussion

The sequencing of plant genomes and transcriptomes has led to the rapid identification of genes and their functional characterization. The transcriptomes are especially helpful in the identification of temporal and spatially differentially expressed genes from plants whose genomes are yet to be sequenced. Also, comparative genomic approaches are a great tool in working with less explored genomes. Availability of the complete genomic sequence for *Arabidopsis* and rice facilitated the identification of a total of six SuSy genes in each of the two plants, representing the entire SuSy gene members of dicots and monocots. The number of SuSy genes in most of the plants could not be exactly determined due to the inadequate coverage of genome sequencing. Sugarcane a crop that accumulates high levels of sugar is expected to have more distinct SuSy genes than *Arabidopsis*, rice or any other crop. Previously five distinct genes encoding different types of sugarcane SuSy isoforms have been characterized at the genomic level with the help of sorghum sequences [18]. Our present work using the long-read, isoform specific transcriptome database searching brings the number of presently known members of the SuSy gene family in sugarcane to at least seven, similar to sorghum, rice, maize, cotton, etc. although other gene families (3, 5 and 6) are yet to be identified. As no adequate information is available for SuSy transcripts in sugarcane the studies of their gene structure, evolutionary relationship as well as their expression patterns provide an important step towards understanding their possible functions in different growth stages and in the composition of sugar and fibre. In a crop like sugarcane, this could get further complex, as there are reportedly 8–14 alleles for a gene and highest sucrose contents as storage sugar.

In duplicated gene families, the homologous genes are reported to have conserved exon intron structure despite having low sequence conservation which can be applied to study their evolutionary relationships [66, 67]. In the present work, comparative screening of SuSy genes revealed that the number and position of introns was highly conserved among SuSy genes in sugarcane and other related monocot plant species. The SuSy genes 1, 2, 4 and 7 identified in this study had distinct gene structures with exon numbers ranging from 14 to 21 (SuSy1–14 exons, SuSy2–16 exons, SuSy4–14 exons and 7–21 exons), interrupted by multiple introns. The availability of full-length reference gene for SuSy2 helped identify exon-intron structure and intron retentions (or pre-mature mRNA transcripts) in certain transcripts which was not observed in other SuSy genes due to the lack of such longer, complete reference genes. The full-length sequences without the use of assembling may be probable isoforms of the gene which were not identified in the previous studies. Within the SuSy gene family, the transcripts had 95–100% similarity at the nucleotide and protein levels. The Ka/Ks ratios for the transcripts of SuSy genes were < 1 revealing that these genes evolved under negative selection that can remove deleterious mutations and result in stabilization of genes. This has probably ensured that the SuSy gene sequences have been conserved across evolutionary history of sugarcane, making them essential for growth and development. The Ka /Ks analysis further showed that the SuSy genes 1, 2, 4 and 7 were highly similar to each other with more than 60% similarity except for SuSy7 which had a slightly less similarity with others (58–59.5%, Additional file 1: Table S9). Another interesting feature observed in SuSy transcripts is the presence of long 5’UTR regions especially in the SuSy1 and 2 gene transcripts. The 5′ UTR region was found to be as long as 3000 bp in some of the SuSy2 transcripts (Additional file 2: Figure S9). The presence of a long 5′ UTR in the SuSy genes was reported in many crop plants [34, 68–70] and differences in the 5′-UTRs and 3′-UTRs among SuSy gene family members was reported in poplar [71] however in sugarcane, this is the first report of such occurrence. The functional significance of this UTR region is not well characterized in any of the crops though a few reports studied the importance of the long 5′ and 3’UTRs in the localization or tissue specific expression of SuSy4 transcripts in potato [72]. The results obtained from NCBI-CDD and InterProScan tools indicated two domains [73], the glycosyl transferase and sucrose synthase domains characteristic of SuSy proteins, however varying in the position of residues among the four different SuSy proteins. Some of the SuSy transcripts had only glycosyltransferase domain (full-length coding sequence with start and stop codons) and the functionality and the regulation of these transcripts are yet to be studied.

A phylogenetic tree was constructed for the SuSy genes identified in this study along with the closely related SuSy gene sequences from sorghum, maize and rice (for an evolutionary relationship among these crop species, please refer review [74]). Characterization of SuSy genes other than 1, 2 and 4 are not yet reported for sugarcane. The UniProt database had a protein sequence for sucrose synthase from *Saccharum* hybrid R570 (accession number AGT16515) which when analysed was found to be closely related to SuSy5 of maize. This was included in the phylogenetic analyses as there was no SuSy5 sequence from sorghum. Similarly, there is a sequence available in NCBI under accession number KF184934 (*Saccharum* hybrid cultivar R570, cultivar R570 clone BAC 119B13, 4 ordered pieces, sequencing in progress), the protein sequence of which is 95% similar to SuSy7 of sorghum and SuSy7 transcript identified in this study. However, this was included in the phylogenetic analysis (labelled as “X”, Fig. 3a, Additional file 2: Figure S10). Exon/intron gene structures to an extent help in the prediction of origin, relationships and possible function of different SuSy genes [7]. The SuSy genes of angiosperms could be subdivided into three distinct subgroups, arbitrarily designated as class I, II and III [18, 75]. The phylogenetic tree in our study clearly distinguished the members into two separate clades. The clade one was sub divided into two which had two sub-clades of SuSy sequences. One sub-clade was composed of SuSy1 and 2 sequences. The other sub-clade was composed of SuSy3 and 4, while the other clade was formed by SuSy5, 6 and 7 sequences suggestive of three classes of SuSy genes. Phylogenetic analysis of cotton SuSy genes and other plant homologues classified the SuSy genes into three distinct families as Sus I, II and III, respectively [18]. Such a classification is currently lacking in sugarcane as many of the SuSy gene members are yet to be characterized. In sugarcane, sorghum, rice and maize, SuSy1 shares more than 90% similarity with SuSy2 while SuSy5 and 6 shares 80% similarity, with the variation mainly present in the C terminal and N terminal regions of the proteins (Additional file 2: Figure S11). The N terminal variations might be attributed to different localization signals [76] while C terminal is reported to be highly variable in SuSy genes [77]. This further complicates the identification of SuSy genes. Even for a well-annotated crop genome like the one of maize*,* inconsistencies occur in the nomenclature of the SuSy genes due to the high similarity existing between/among the isoforms/transcript variants/gene family members as can be seen when a search is made for SuSy genes in the NCBI-GenBank or UniProt databases. This is the case with sugarcane and sorghum SuSy genes found in the public databases. In rice, SuSy5 and SuSy7 were found to be near identical with only nine SNPs between their sequences and they were found to be near to each other on chromosome 4 [34]. This close relationship between SuSy5 and 7 can be observed in the phylogenetic tree especially for rice and maize (Fig. 3a). The MEME suite identified motif positions for each member that were highly conserved without any insertion or deletion. The distribution of motifs throughout the sequences is highly conserved and only when the set limit for motif prediction was above 25, variations could be observed among the SuSy genes. This could indicate that SuSy proteins have the potential to recognize the same target genes with similar or overlapping functions in vivo [12]. However, it is also possible that different SuSy genes may have distinct, non-overlapping functions within the same cell [78].

In order to understand the potential functions of specific SuSy genes expressed in sugarcane transcriptome, the tissue-specific expression of SuSy genes were examined in various tissues including leaves, top, middle and bottom internodes of the sugarcane culm, and root in addition to the culm transcriptomes from progenitors’ species *S. spontaneum* and *S. officinarum*. The spatial expression pattern of the four SuSy genes 1, 2, 4 and 7 was qualitatively studied from the RNA-Seq reads in the above tissues from two sugarcane genotypes, independently. All four genes were highly differentially expressed between the top and bottom tissues with the top tissue having very high expression of SuSy genes. SuSy1 showed high expression levels in top internode and root tissues, while the leaves, and the middle and bottom internodes had very little expression. SuSy2 and SuSy7 showed similar patterns in top and root tissues, while moderate expression levels were observed in the other tissues. SuSy4 did not show any tissue specific expression, however showed higher expression in the leaves than the other genes.

Overall, the top tissue is metabolically active compared to the bottom and shows higher expression of SuSy genes indicating a strong role for SuSy during the phase of growth where carbon is largely partitioned towards fibre synthesis, respiration and non-sucrose storage functions [79] . The middle internode where metabolism is largely directed towards sucrose accumulation had moderate expression. The leaves (source tissue) showed very low expression levels. The roots which are another strong sink displayed very high levels of SuSy similar to that of the top internodes. The expression profiles were consistent in both the genotypes, across all the tissues used for the analyses. Root expression (other than starch storage organs like carrot, sugarbeet) of SuSy genes have been reported earlier in rice [31], wheat and maize, however under hypoxia [80, 81]. These two tissues represent the major sinks and our data strongly supports SuSy playing a major role in driving sink strength. This in turn elevates the importance of SuSy from partitioning to driving biomass accumulation. SuSy activity was reported to be related to total sugar accumulation rate in sugarcane [82]. Sink strength is the key factor in influencing biomass accumulation and SuSy is reported to be a biochemical determinant of sink strength in developing tomato fruits [83, 84] and in potato tubers [73]. Among the SuSy genes studied, SuSy1 and SuSy2 were highly expressed in the two strong sink tissues (top internodes and root tissues), while SuSy4 and SuSy7 were expressed in all the tissues probably indicating a ‘house-keeping’ role for the latter. SuSy7 is a new isoform that was not reported earlier in sugarcane. In rice, an expression profile analysis of SuSy transcripts, including SuSy7 revealed that they were abundantly detected in sink tissues such as roots, flowers, and immature seeds [34]. This gene showed highest expression in *S. officinarum* while other isoforms showed moderate expression levels in both the progenitors probably indicating their sub-genomic origin.

The high expression levels of SuSy genes in the strong sinks is consistent with SuSy playing a major role in sucrose breakdown to provide substrate for cell wall synthesis, and provide precursors under energy conditions where available oxygen might be limiting (roots). In another study, the expression of SuSy was highly correlated with sucrose content **(**Additional file 2: Figure S12**)** with the high sugar genotypes showing high expression of SuSy genes [85].

## Conclusion

The role of the SuSy genes is complex as SuSy is generally reported to be hydrolytic rather than operating in the synthesis direction. The high expression in sink tissues may be associated with the production of UDP-glucose to support cell wall biosynthesis in these growing tissues. Further expression studies at different developmental stages would help in elucidating the role of SuSy genes in determining the accumulation of sucrose and fibre in sugarcane. It should be noted that, although our efforts in this study, through deep sequencing and database searching, have brought the count of sugarcane SuSy gene family members to seven, we could also expect possibilities of other additional yet to be identified paralogues. More information, especially the chromosomal location of the SuSy genes in sugarcane, is needed to determine a more precise evolutionary relationship among these SuSy genes. To date, although there are few studies on SuSy genes in sugarcane, there is no systematic functional analysis or expression studies for the entire SuSy gene family. We have demonstrated differential expression of the SuSy genes in sugarcane, with respect to sink strength, tissue specificity and sub-genomic origins.

## Additional files

Additional file 1: **Table S1.** Details of RNA-Seq reads from various tissues used for the SuSy expression study. **Table S2.** Raw counts for SuSy gene expression from different tissue sample. **Table S3.** SuSy transcripts from the SUGIT transcriptome database. **Table S4.** Codon-based Test of Positive Selection for analysis between SUSY1 sequences. **Table S5.** Codon-based Test of Positive Selection for analysis between SUSY2 sequences. **Table S6.** Codon-based Test of Positive Selection for analysis between SUSY4 sequences. **Table S7.** Log2-fold change values and FDR corrected heat map for SuSy transcripts expression in the genotype KQ228. **S8.** Log2-fold change values and FDR corrected heat map for SuSy transcripts expression in the genotype Q208. **Table S9.** Similarity percentage observed between different SuSy isoforms using Clustal W 2.1 (XLSX 362 kb)

Additional file 2: **Figure S1.** Identification and sequence retrieval of SuSy transcripts from the SUGIT transcriptome reference database. Shown here is the reads mapping to the reference SuSy4 gene from sugarcane using large gap mapping tool in the CLC-WB v 10. **Figure S2.** Alignment of nucleotide sequences of the SUGIT-SuSy1 transcripts with the reference sequences using Geneious v.11. Reference genes used in the alignment are from Sorghum accession numbers NC_012870, XM_002465116; bottom with *Zea mays* NC_024467 and NM_001111941. **Figure S3.** Alignment of nucleotide sequences of the SUGIT-SuSy2 transcripts with the reference sequences using Geneious v.11. Reference genes for alignment is from *Saccharum officinarum*, accession number AY118266 and its mRNA and cDNA sequences. **Figure S4.** Alignment of nucleotide sequences of the SUGIT-SuSy4 transcripts with the reference sequences using Geneious v.11. Reference genes used for alignment are from *Zea mays* accession numbers NC_012870 and mRNA and cDNA sequences of accession XM_2465268. **Figure S5.** Alignment of nucleotide sequences of the SUGIT-SuSy7 transcripts with the reference sequences using Geneious v.11. Reference genes used are from *Zea mays* accession numbers NC_024463 and mRNA and cDNA sequences of accession number XM_008646897. **Figure S6.** Conserved exon-intron structure across the monocots species available in Phytozome. Using BLAST tool available in the GBrowse, the SuSy transcripts were annotated with Sorghum as the reference genome. **Figure S7.** a. In genotype KQ228 SuSy 1, 2 and 7 have significantly different expression between each of the tissue types, based on one -way ANOVA results *p* < 0.01. b. Based on tukey t-test results the expression rates of SuSy 1, 2 and 7 are significantly higher in root and top internode tissue. Analysis undertaken in SPS stats v. 23. **Figure S8.** a. In genotype Q208 SuSy 1, 2 and 7 have significantly different expression between each of the tissue types, based on one -way ANOVA results p < 0.01. b. Based on tukey t-test results the expression rates of SuSy 1, 2 and 7 are significantly higher in root and top internode tissue. Analysis undertaken in SPS stats v. 23. **Figure S9.** The 5′ upstream sequence in one of the SuSy2 transcript spanning to 2243 bp with an ORF length of 2409 bp. **Figure S10.** Multiple sequence alignment of SuSy gene sequences used for phylogenetic analysis. **Figure S11.** Variations present in the C terminal (A) and N terminal regions (B) of the identified SuSy1, 2, 4 and 7 proteins. The N terminal variations might be attributed to different localization signals while C terminal is reported to be highly variable among the SuSy genes [81]. **Figure S12.** The high expression level of SUGIT SuSy gene transcripts coincident with the high levels of sucrose and fiber in sugarcane hybrid genotypes. Heat maps showing log2-scaled RPKM (reads per kilobase per million reads) values for expression profiles of SuSy genes 1, 2, 4 and 7 in the sugarcane hybrid genotypes (XLSX 19426 kb)

## Abbreviations

CDD: Conserved domain database
cDNA: Complementary DNA
GDP: Guanosine diphosphate
GTP: guanidine tri phosphate
mRNA: Messenger RNA
NADP: Nicotinamide Adenosine diphosphate
NCBI: National Center for Biotechnology Information
NGS: next generation sequencing
Nr: database: Non-redundant database
ORF: Open reading frame
RNA-seq: Ribonucleic acid sequencing
RPKM: Reads per kilobase per million mapped reads
SNP: single nucleotide polymorphism
SRA: Sugar Research Australia
SUGIT: sugarcane Iso-Seq transcriptome database
SuSy: sucrose synthase
UDP: uridine diphosphate
UTR: Untranslated region
